# Extracellular ATP Regulates CD73 and ABCC6 Expression in HepG2 Cells

**DOI:** 10.3389/fmolb.2018.00075

**Published:** 2018-08-14

**Authors:** Fabio Martinelli, Flavia Cuviello, Maria C. Pace, Maria F. Armentano, Rocchina Miglionico, Angela Ostuni, Faustino Bisaccia

**Affiliations:** Department of Sciences University of Basilicata, Potenza, Italy

**Keywords:** ABCC6, Probenecid, purinergic pathway, CD73, HepG2 cells

## Abstract

The ATP-binding cassette sub-family C member 6 transporter (ABCC6) is an ATP dependent transporter mainly found in the basolateral plasma membrane of hepatic and kidney cells. Mutations in *ABCC6* gene were associated to the Pseudoxanthoma elasticum (PXE), an autosomal recessive disease characterized by a progressive ectopic calcification of elastic fibers in dermal, ocular, and vascular tissues. It is reported that the over-expression of ABCC6 in HEK293 cells results in the cellular efflux of ATP and other nucleoside triphosphates, which in turn are rapidly converted into nucleoside monophosphates and pyrophosphate (PPi). Since PPi is an inhibitor of mineralization, it was proposed that the absence of circulating PPi in PXE patients results in the ectopic mineralization, a typical feature of PXE. In the extracellular environment, ATP is converted, not only into pyrophosphate, but also into AMP by an ectonucleosidase, which in turn is transformed into adenosine and phosphate. ABCC6 protein is thus involved in the production of extracellular adenosine and therefore it could have a role in the activation of the purinergic system. In the liver, purinergic signaling has been shown to regulate key basic cellular functions. Our previous studies showed that in *ABCC6* knockdown HepG2 cells the expression of some genes, related with the calcification processes, is dysregulated. In this study, experiments have been carried out in order to verify if ABCC6, besides supplying the pyrophosphate required to prevent the mineralization of soft tissues, also plays a role in the activation of the purinergic system. For this purpose, the transport activity of ABCC6 was blocked with Probenecid and the expression of ABCC6 and NT5E was analyzed with real time PCR and western blotting. The results of this study showed that both proteins are downregulated in the presence of Probenecid and upregulated in the presence of adenosine or ATP.

## Introduction

**Scheme 1:**
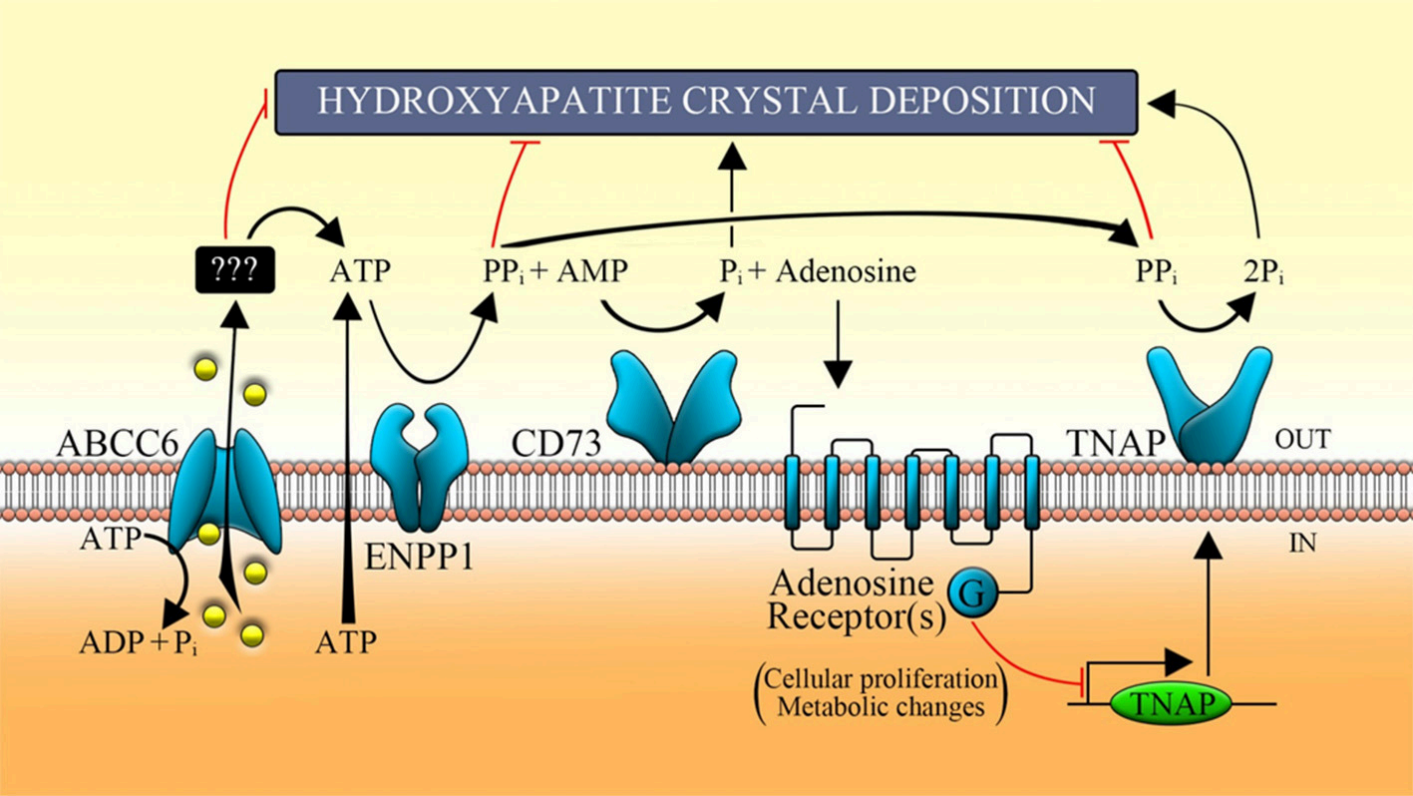
Function of the main proteins involved in the purinergic pathway. ABCC6 transporter allows, in part, the release of ATP, which is metabolized by some ecto-nucleotidases (such as ENPP1) in AMP, in adenosine by CD73 and Pi by TNAP. ABCC6, ATP-binding cassette, sub-family C, member 6; ENPP1, ecto-nucleotide pyrophosphatase/phosphodiesterase type I; CD73, cluster of differentiation 73; TNAP, tissue non-specific alkaline phosphatase; Pi, inorganic phosphate; PPi, inorganic pyrophosphate.

The ectopic mineralization of the soft tissues represents a pathological condition characterized by the deposition of calcium phosphate complexes in the soft connective tissues. Inorganic pyrophosphate (PPi) acts as a powerful inhibitor of mineralization, while phosphate (Pi) is a pro-mineralization factor, then an appropriate PPi/Pi ratio is essential for preventing the ectopic mineralization under homeostatic conditions (Terkeltaub, 2001). The pathophysiology of hereditary ectopic calcifications has been extensively studied in three rare diseases, namely

pseudoxanthoma elasticum (PXE, OMIM 264800), generalized arterial calcification of infancy (GACI, OMIM 208000) and arterial calcification due to deficiency of CD73 (ACDC, OMIM 211800). Scheme 1 shows how the ATP released from cells is converted by a protein encoded by an ecto-nucleotide pyrophosphatase/phosphodiesterase 1 gene (*ENPP1*) into PPi and AMP, and the latter into adenosine and phosphate by an extracellular nucleotidase known as CD73. PPi is a potent mineralization inhibitor, while adenosine is a purinergic pathway activator. Mutations of *ABCC6* (ATP-binding cassette, sub-family C, member 6) gene causes PXE an autosomal recessive disorder characterized by calcified and fragmented elastic fibers of the skin, the retina and the vascular wall (Favre et al., 2017; Germain, 2017). The ABCC6 encoded protein containing an additional NH2-terminal transmembrane domain is expressed essentially in liver and kidney (Ostuni et al., 2010; Lee et al., 2014; Cuviello et al., 2015; Miglionico et al., 2016). Recently, it has been suggested that, in pathological conditions, the loss of the ABCC6-dependent outflow of ATP would result in an alteration of the extracellular PPi/Pi ratio with consequent mineralization processes (Jansen et al., 2013, 2014). However, while the effects of the lack of PPi production in the extracellular space are clear, the effects caused by the lack of production of adenosine when *ABCC6* is mutated are not yet clear. The production of pyrophosphate is thus the result of the sequential action of ABCC6 and ENPP1 and explains the histopathological similarity between PXE and GACI diseases. Indeed, GACI is a rare autosomal recessive disease, caused by mutations of *ENPP1*, characterized by severe calcification of the elastic lamina inside the large and medium-sized arteries leading to arterial stenosis and heart failure within the first months of life (Rutsch et al., 2001, 2003). The AMP produced by ENPP1 is in turn transformed in adenosine and phosphate by the CD73 protein encoded by the *NT5E* gene whose mutations cause ACDC (St. Hilaire et al., 2011). ABCC6 protein is thus involved in the production of extracellular adenosine and therefore could be involved on the activation of the purinergic system. In this paper the term purinergic pathway will be used to indicate any activity related to the presence of extracellular nucleotides and purine nucleosides. However, although this scheme has been shared by various research groups and pyrophosphate has been proposed as a drug to treat PXE (Pomozi et al., 2017; Kranenburg et al., 2018), various aspects have not yet been clarified. First of all, it is not currently known by what mechanism this transporter promotes the release of ATP. In fact, it is not yet defined whether ABCC6 directly transports ATP and whether there is a substrate transported by ABCC6 other than ATP (Jansen et al., 2013). Moreover, it is not clear whether ABCC6 contributes to the activation of the purinergic system, which is able to regulate key basic cellular functions such as glucose/lipid metabolism, protein synthesis, and ionic secretion, also affecting homeostatic processes (cell cycle, inflammatory response, immunity, and mineralization; Fausther and Sévigny, 2011; Burnstock, 2012, 2017; Tapper et al., 2012; Ziegler et al., 2017).

Our previous studies, aimed at understanding the role of ABCC6 in cells that physiologically express it, have shown that *ABCC6*-silenced HepG2 cells showed changes in the cell phenotype as a consequence of a gene expression dysregulation and of the cell cycle alteration, suggesting that this protein could have another role besides promoting the PPi accumulation outside the cell (Miglionico et al., 2014, 2017). In this study, a panel of experiments have been performed to verify whether the ABCC6 is involved in the purinergic pathway.

## Materials and methods

### Cell culture and treatments

Human embryonic kidney cells 293 (HEK293) and human hepatocellular carcinoma cells (HepG2) were maintained in Dulbecco's modified Eagle's medium (DMEM) containing 4.5 g/L glucose, supplemented with 10% fetal bovine serum (FBS), 2 mM L-glutamine, penicillin (100 U/mL), and streptomycin (100 mg/mL) at 37°C, in an atmosphere humidified with 5% of CO_2_. HepG2 cells were seeded in 6-wells plates (6 × 10^5^ cells/well) and treated for 48 h with Probenecid, ATP, adenosine at the indicated concentrations. Probenecid was dissolved in dimethyl sulfoxide (DMSO) at 30 mg/ml as stock solution which was then diluted with DMEM to the desired concentrations. The final concentration of DMSO did not exceed 1% v/v. Control cells were treated at the same final percentage of DMSO. All compounds were purchased from Sigma-Aldrich (unless otherwise indicated).

### Stable transfection of *ABCC6*

HEK293 cells were grown to 50–70% confluency and then transfected at 3:1 ratio FuGene 6 transfection reagent (Promega): DNA using Flag-pcDNA vector (as control) or Flag-pcDNA containing sequence coding for ABCC6 (*ABCC6*-Flag-pcDNA vector). Limiting dilutions were used to select individual clones. For 18 days medium containing geneticin (800 μg/mL, Euroclone) was changed in cultures twice weekly. As clones grew, a lower amount of G418 for maintenance (400 μg/mL of G418) was used.

### Doxorubicin efflux assay

HEK293 cells stably over-expressing ABCC6 and control cells (3 × 10^5^ cells/well) were seeded in 12-wells plates, pre-treated with poly-D-lysine hydrobromide for 30 min, and allowed to adhere for 24 h. After incubation with 1 mM Probenecid in PBS for 1 h, 5 μM doxorubicin was added to each well and cells were incubated in the dark for 30 min at 37°C in 5% of CO_2_. Cells incubated only with PBS were used as negative control. Cells were, then, washed two times with cold PBS and external fluorescence was measured at different times using a black 96-wells plates and GloMax Multi Detection System with blue filter (ex. 490 nm, em. 510–570 nm).

### Viability assay

The MTT (3-(4, 5-dimethyl thiazol-2yl)-2, 5-diphenyl tetrazolium bromide) assay was used to assess cell viability. HepG2 cells, seeded at a density of 5 × 10^3^/well in 96-wells plates, were treated with different concentrations of Probenecid (250, 500, and 1,000 μM) for 24 and 48 h. Cells were then incubated with fresh medium containing 15% MTT and incubated for 4 h at 37°C. The formazan crystals were finally dissolved for 1 h at 37°C in DMSO:isopropanol (1:1) solution with 1% of Triton X-100. MTT reduction was quantified by measuring the light absorbance at 570 nm, with background subtraction at 630 nm, using a microplate reader (Multiskan^TM^ GO Microplate Spectrophotometer, Thermo Scientific). Results were presented as percentage of the control (cells treated only with vehicle DMSO), defined as 100% of cell viability. Each test was repeated three times in triplicate. Cells were also examined under a phase-contrast microscope and representative fields were photographed using a Nikon Coolpix P6000.

### RT-PCR and real-time PCR

RNA was extracted from HepG2 cells using Quick-RNA MiniPrep kit (Zymo Research), then was transcribed to cDNA using random primers and High-Capacity cDNA Reverse Transcription Kit (Applied Biosystem). cDNA was amplified via real-time PCR using iTaq^TM^ Universal SYBR® Green Supermix (Bio-Rad) on the 7500 Fast Real-Time PCR System (Applied Biosystems). Primers were designed for spanning exon-exon junctions eliminating undesirable genomic DNA amplification: β*-actin*, forward: 5′-CCTGGCACCCAGCACAAT-3′, reverse: 5′-GCCGATCCACACGGAGTACT-3′; *ABCB1*, forward: 5′- CCTTCAGGGTTTCACATTTGG-3′, reverse: 5′-ACTCACATCCTGTCTGAGCA-3′; *ABCC1*, forward: 5′-GCTGATGGAGGCTGACAAGG-3′, reverse: 5′-GATGCTGAGGAAGGAGATGAAGAG-3′; *ABCC2*, forward: 5′-CCCTTGTCCTGGAAGATGTT-3′, reverse: 5′-AGAGCCTTCATCAACCAGG-3′; *ABCC3*, forward: 5′-CCACACCACAACCACCTTCAC-3′. reverse: 5′-CTCGGCGTCCAGCACATTG-3′; *ABCC4*, forward: 5′-GCACACCAGGATTTACATTCAGAG-3′, reverse: 5′-CCAGACGGACGGCAAACC-3′; *ABCC5*, forward: 5′-CCACCATCCACGCCTACAATAAAG-3′, reverse: 5′-ACAGCCAGCCACCGCATC-3′; *ABCC6*, forward: 5′-AAGGAACCACCATCAGGAGGAG-3′, reverse: 5′-ACCAGCGACACAGAGAAGAGG-3′; *ABCG2*, forward: 5′-ATCACTGATCCTTCCATCTTG-3′, reverse: 5′-GCTTAGACATCCTTTTCAGG-3′. The comparative threshold cycle method (ΔΔCt) was used to quantify relative amounts of product transcripts with β-actin as endogenous reference control (Livak and Schmittgen, 2001). The specificity of amplicons was confirmed by melting-curve analysis. Each test was repeated three times in triplicate.

### Western blot analysis

HepG2 cells were lysed in Laemmli sample buffer (60 mM Tris–HCl pH 6.8, 10% glycerol, 2% SDS, 1% β-mercaptoethanol and 0.002% bromophenol blue) supplemented with proteases and phosphatases inhibitors cocktail. Subsequently, the cellular lysis is accomplished by three thermal shock to denature proteins within the cell membranes. Finally, the lysates were centrifugated at 4,000 rpm for 2 min and the proteins were resolved on 8 or 15% SDS-PAGE gels. After electroblotting on nitrocellulose or PVDF membranes (Amersham Protran, GE Healthcare Life Sciences), membranes were blocked for 1 h with 0.25% non-fat milk in PBS-T pH 7.4 and incubated overnight at 4°C with specific primary antibodies: 1:400 anti-β-actin diluted in PBS-T with 5% non-fat milk and anti-ABCB1 1:100 diluted in PBS-T with 0.25% non-fat milk (Abcam); 1:100 anti-ABCC6 (H-70); and 1:100 anti-CD73 (IE9) diluted in PBS-T with 0.25% non-fat milk (Santa Cruz Biotechnology). Membranes were washed three times for 10 min with PBS-T and incubated with appropriate horseradish peroxidase-conjugated secondary antibody at room temperature for 1 h. The membranes were washed three times for 10 min with PBS-T and signals visualized by Chemiluminescent Peroxidase Substrate-1 or Super Signal West Femto Maximum Sensitivity Substrate (Thermo Scientific), using Chemidoc™ XRS detection system equipped with Image Lab Software for image acquisition (BioRad). Densitometric analysis was performed by using ImageJ software (National Institute of Health, Bethesda, MD). The protein expression level in control sample was taken as 100%. Each result was expressed as percentage of the value of control sample. Each test was repeated three times.

### Statistical analysis

Data are presented as mean ± SD. Student's *t*-test was performed pairwise to compare control and treated samples. Differences were considered significant whenever *p*-value <0.05. Statistical analysis was performed using statistical GraphPad software.

## Results

In this study, we aimed to verify whether gene dysregulation, previously observed in *ABCC6*-silenced HepG2 cells (Miglionico et al., 2014), was mediated by the purinergic system. For this purpose, the ABCC6 activity in HepG2 cells was inhibited with Probenecid, a known ABC transporters inhibitor (Silverman et al., 2008; Ma et al., 2009), and the effect of adenosine and ATP was evaluated on CD73 expression.

In order to verify the effective inhibition of Probenecid on ABCC6 transport activity, we evaluated its effect on the efflux of doxorubicin, a known substrate also carried by ABCC6, from stably ABCC6-overexpressing HEK293 cells. The efflux of doxorubicin is significantly higher compared with mock transfected cells, suggesting that ABCC6 contributes to the release of doxorubicin (Supplemental Figure S1). Moreover, pretreating both cell cultures with 1 mM Probenecid, no significant differences in the doxorubicin efflux were observed, suggesting that Probenecid inhibited the transport activity carried out by ABCC6.

**Figure 1:**
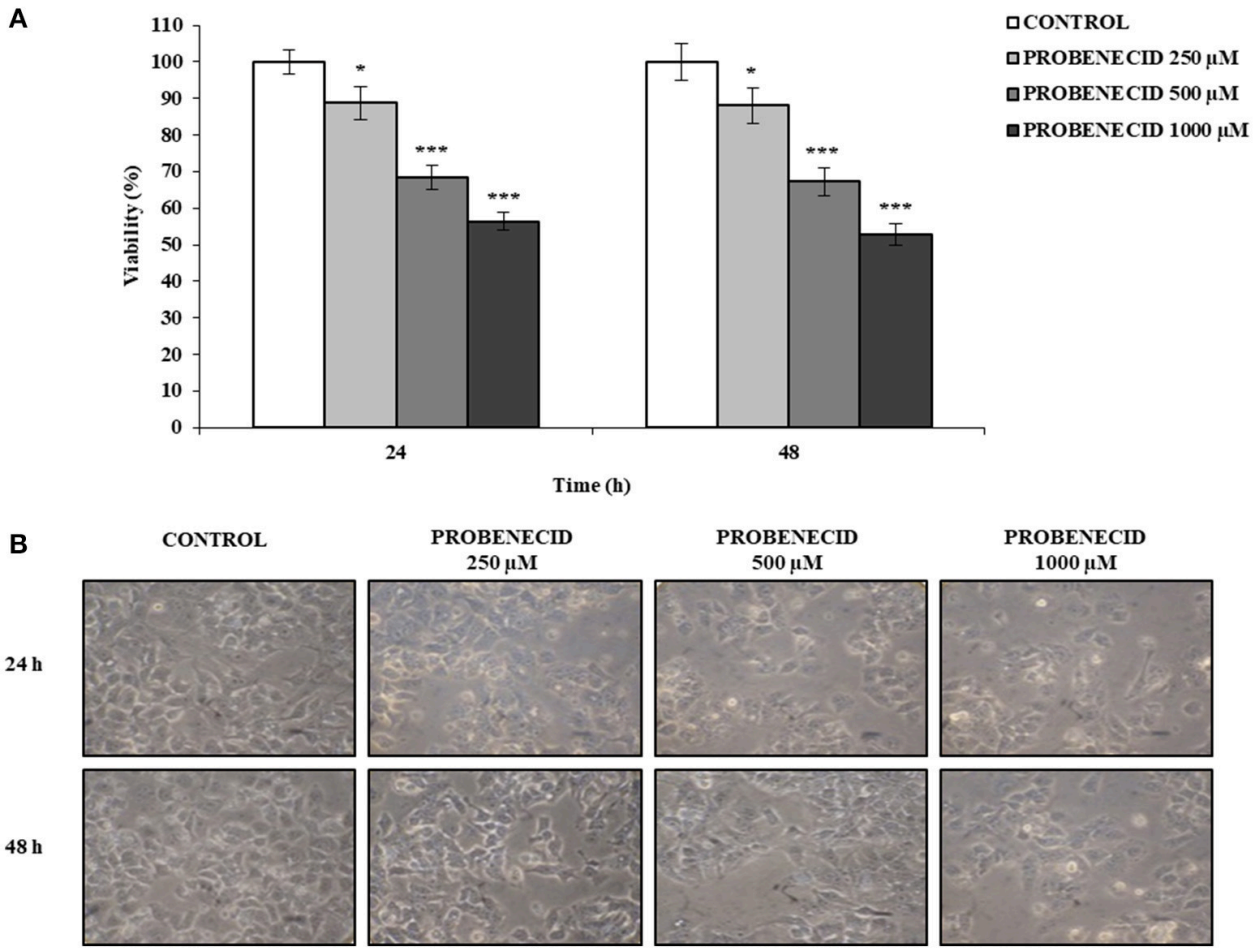
Effects of Probenecid on viability and morphology of HepG2 cells. **(A)** Cells were treated with Probenecid at different concentrations (250, 500, and 1,000 μM) for 24 and 48 h. Data are expressed as a percentage of the control group and presented as the means ± SD of three replicates from three independent experiments. *p < 0.05, ***p < 0.001, Student's *t*-test. **(B)** Representative images of Probenecid-treated HepG2 cells. DMSO-treated cells were used as control.

Using the MTT viability assay, the dose-dependent effects of Probenecid were tested on HepG2 cells, treated for 24 and 48 h. Probenecid did not exhibit significant cellular toxicity up to 250 μM for 48 h (Figure 1A). Probenecid-treated HepG2 cells did not show morphological alterations (in terms of the number of vacuoles and shape) compared to untreated HepG2 cells (Figure 1B).

**Figure 2:**
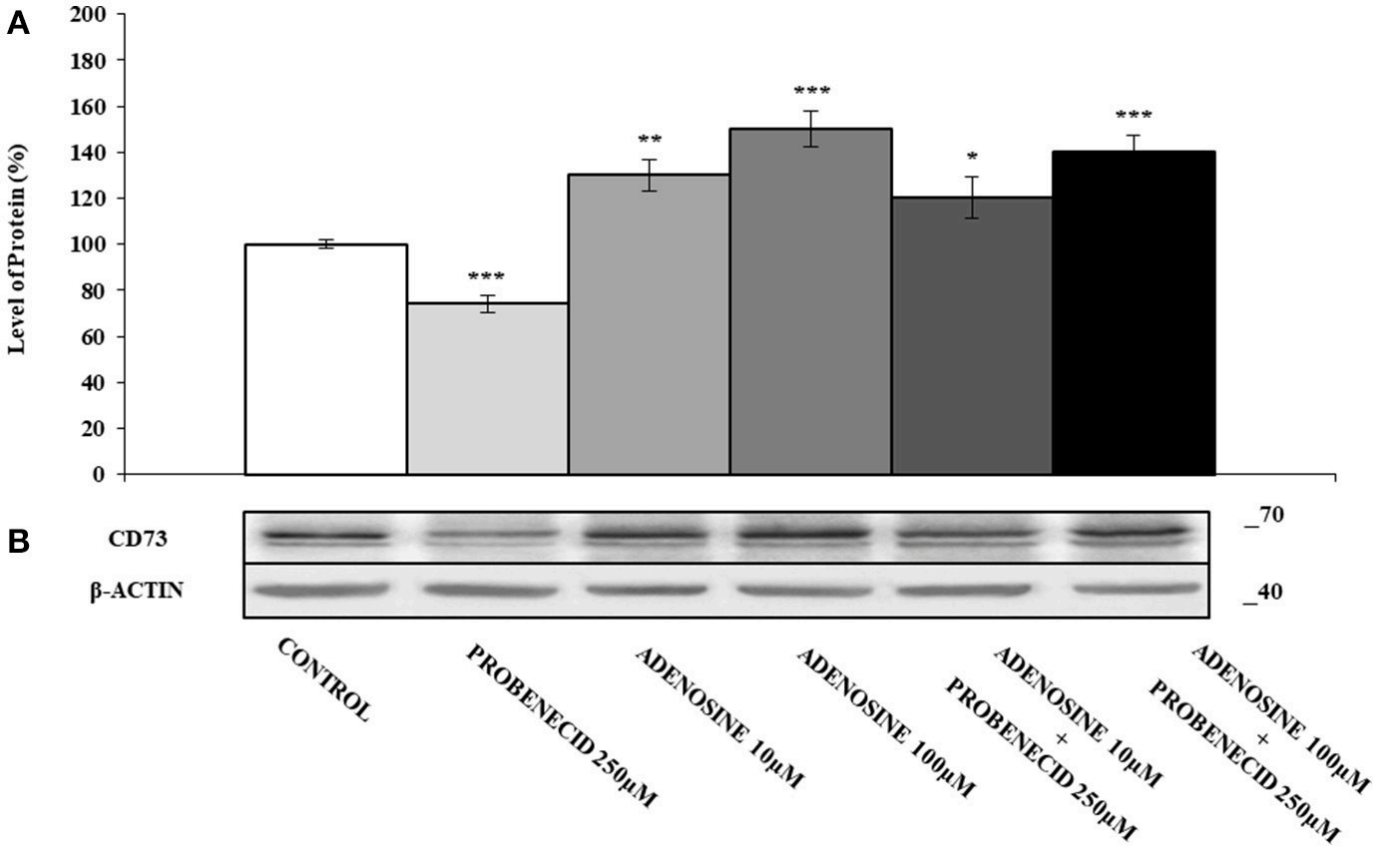
The effects of adenosine on CD73 protein expression, with or without Probenecid. **(A,B)** Densitometric analysis and representative western blot of CD73 in three independent experiments (means ± SD). *p < 0.05, **p < 0.01, ***p < 0.001, Student's *t*-test. β-actin was used as a loading control. All the experiments were performed treating HepG2 cells with adenosine and/or Probenecid for 48 h. DMSO-treated cells were used as control.

**Figure 3:**
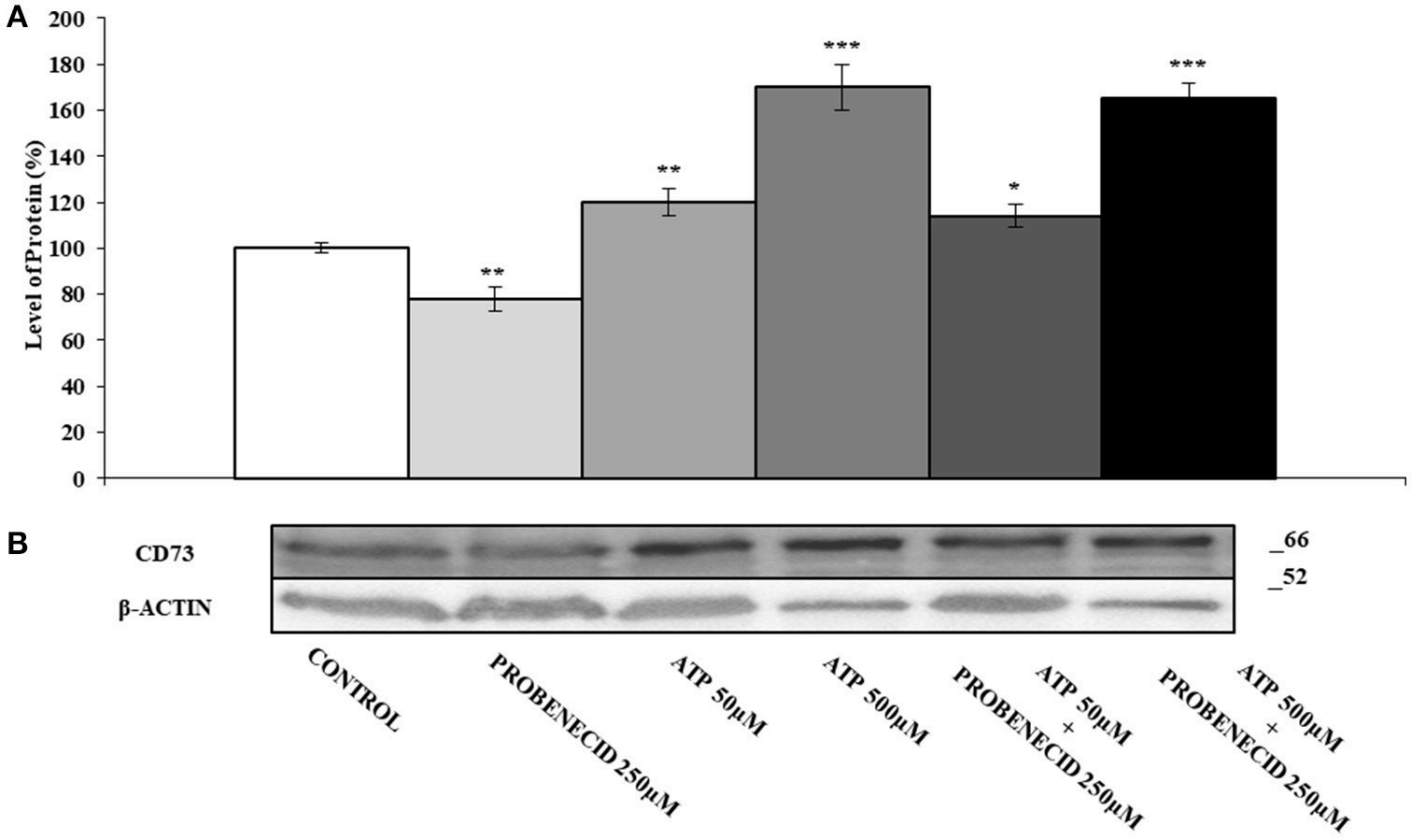
The effects of ATP on CD73 protein expression, with or without Probenecid. **(A,B)** Densitometric analysis and representative western blot of CD73 in three independent experiments (means ± SD). *p < 0.05, **p < 0.01, ***p < 0.001, Student's *t*-test. β-actin was used as a loading control. All the experiments were performed treating HepG2 cells with ATP and/or Probenecid for 48 h. DMSO-treated cells were used as control.

In Figures 2A,B the effect of the ABCC6 inhibition on CD73 expression is shown. CD73 protein level was reduced of about 30% after treatment of cells with 250 μM Probenecid for 48 h. Furthermore, CD73 expression increased of 30 and 40% when cells were treated with 10 and 100 μM adenosine, respectively, and did not change even in the presence of Probenecid. As ATP is the substrate carried by the ABCC6, cells were incubated with ATP in the presence or absence of Probenecid. As previously seen on cells treated with adenosine, the expression of CD73 was increased in a dose-dependent manner (20 and 60% with ATP at 50 and 500 μM, respectively), and once again did not change in the presence of Probenecid (Figures 3A,B).

**Figure 4:**
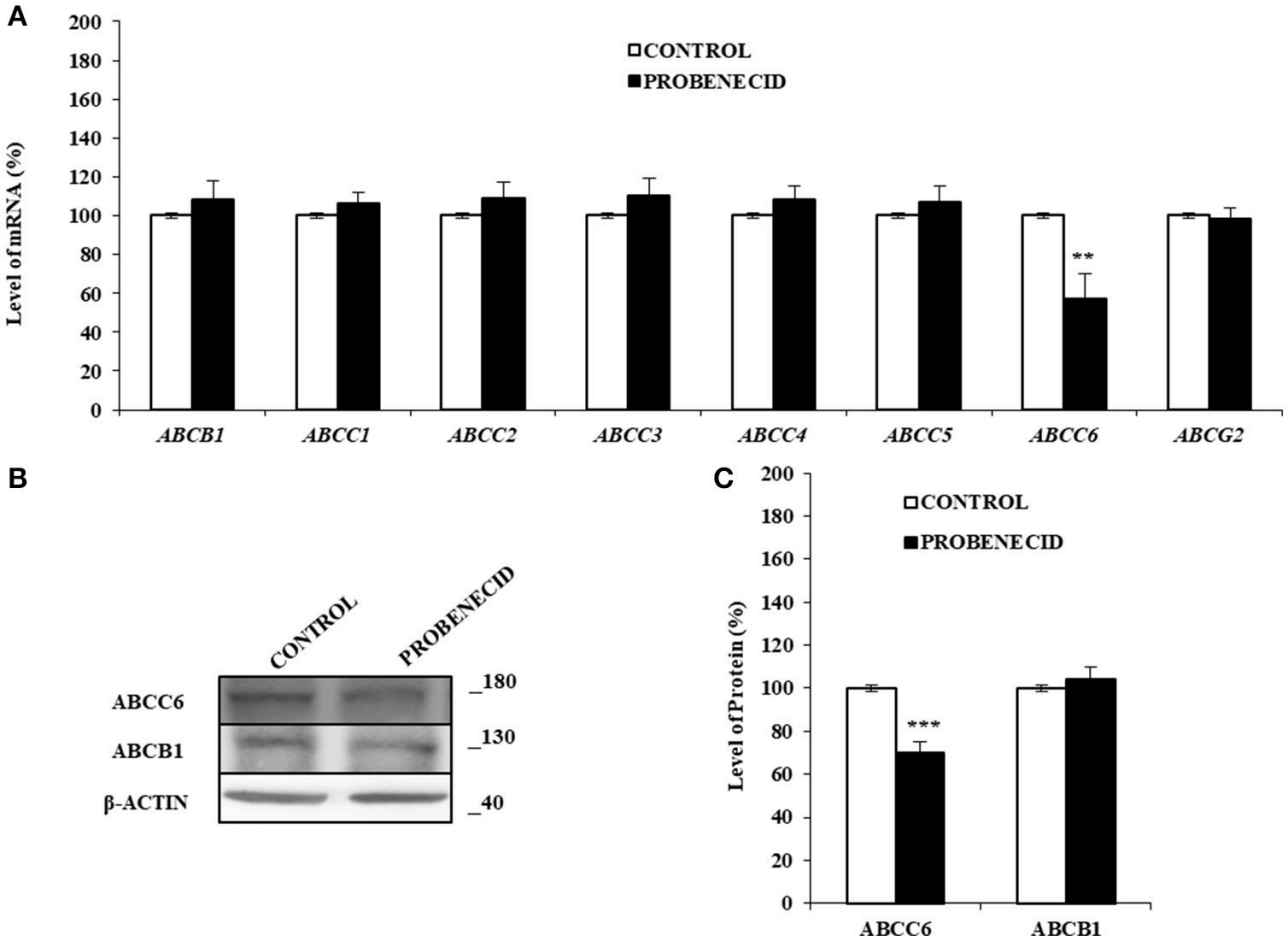
The effects of Probenecid on some *ABC* genes and ABCC6 and ABCB1 proteins. **(A)** Fold change in some *ABCs* mRNA levels compared to the control cells. Results are represented as the mean ± S.D. (*n* = 3). **p < 0.01, Student's *t*-test. **(B,C)** Representative western blot and densitometric analysis of the ABCC6 and ABCB1 in three independent experiments (means ± SD). ***p < 0.001, Student's *t*-test. β-actin was used as a loading control. All the experiments were performed treating HepG2 cells with 250 μM of Probenecid for 48 h. DMSO-treated cells were used as control.

In order to verify whether Probenecid was able to affect the transcript levels of some *ABC* transporters genes, a Real Time-PCR experiment was carried out (Figure 4A). No variation was observed for all the *ABC*s examined except for *ABCC6*, which was significantly reduced (about 30%) in HepG2 cells treated with Probenecid. This result was also confirmed at protein level by western blotting analysis (Figures 4B,C).

**Figure 5:**
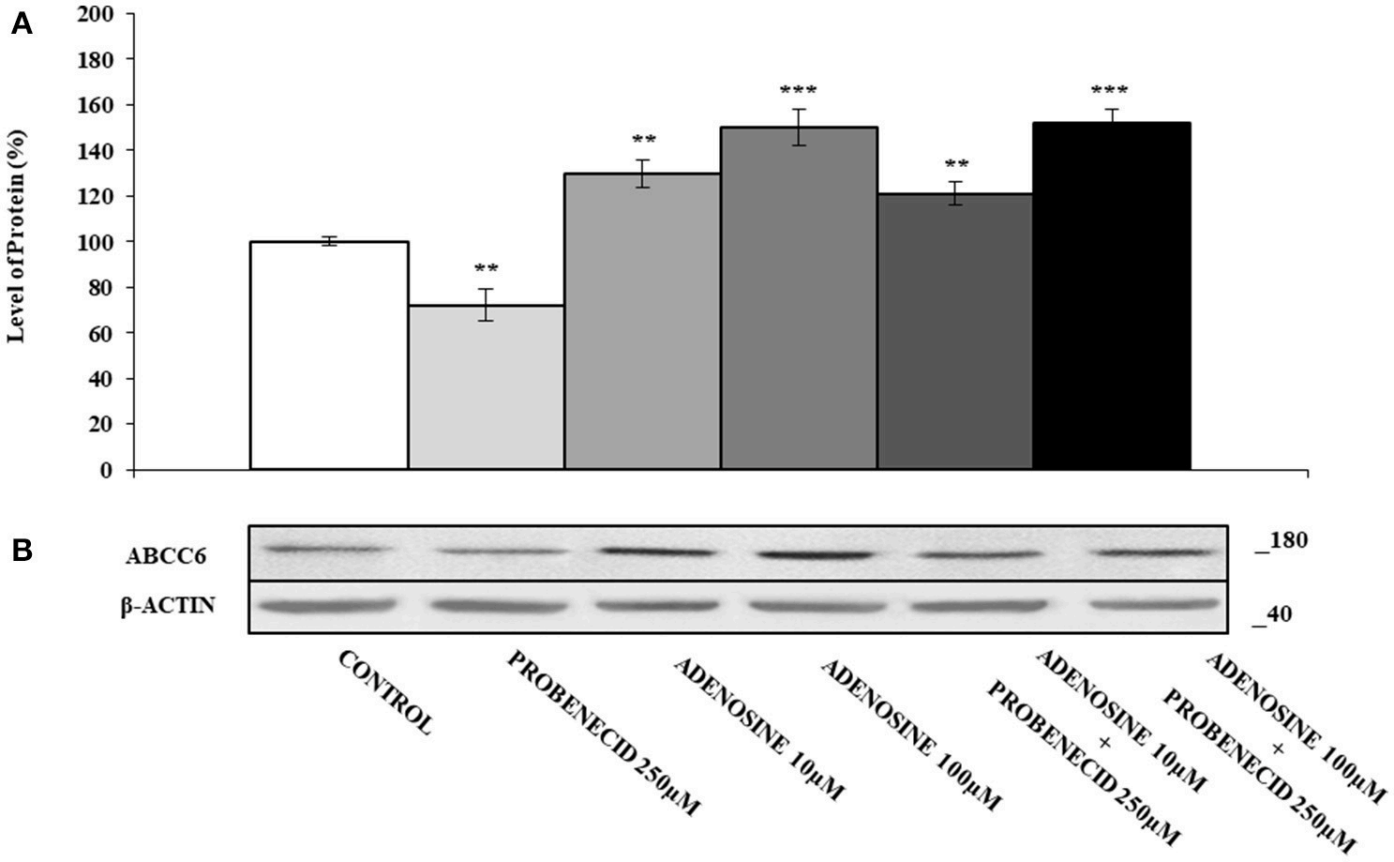
The effects of adenosine on ABCC6 protein expression, with or without Probenecid. **(A,B)** Densitometric analysis and representative western blot of the ABCC6 protein in three independent experiments (means ± SD). **p < 0.01, ***p < 0.001, Student's *t*-test. β-actin was used as a loading control. All the experiments were performed treating HepG2 cells with adenosine and/or Probenecid for 48 h. DMSO-treated cells were used as control.

Figure 5 shows the effect of adenosine on the expression of ABCC6. The addition of different concentration of adenosine (10 and 100 μM) increased the ABCC6 protein level about of 40 and 60%, respectively. In HepG2 cells treated only with Probenecid, the ABCC6 expression decreased by about 30%, but no changes were observed if Probenecid is used in association with adenosine.

## Discussion

PXE is considered a metabolic disease caused by lack of circulating factors able to prevent mineralization. Jansen et al. (2013, 2014) proposed that this factor is the pyrophosphate derived from the ATP released by ABCC6. Although this hypothesis has been widely demonstrated and pyrophosphate is used to prevent soft tissue mineralization in PXE patients, little is known about the role played by extracellular AMP and by adenosine.

Previous studies in our laboratory showed that knockdown of *ABCC6* leads to dysregulation of some genes including *NT5E*, which has been found under-expressed (Miglionico et al., 2014). In order to verify whether this finding was due to the lack of activation of purinergic pathway, in this study we evaluated if CD73 expression was regulated by the extracellular ATP and adenosine.

We demonstrated that Probenecid, an ABC transporters inhibitor, downregulated the expression of CD73, and adenosine increased its expression. Similar results were obtained by adding ATP, an ABCC6 substrate, which ultimately restores the levels of adenosine. Taken together, these results suggested that ABCC6, promoting the efflux of ATP from the cells, supplies the extracellular compartment with both the pyrophosphate, necessary to prevent the calcification, and purine nucleotides and adenosine, crucial mediators of numerous patho-physiological mechanisms (Burnstock, 2012, 2017).

The decrease of ABCC6 and CD73 expression by Probenecid and their increase by adenosine suggested a role for ABCC6 in the regulation of the purinergic system and could explain the overlapping clinical symptoms of ACDC and PXE.

In conclusion, in this study we demonstrated that ABCC6 supplies the extracellular space of nucleosides and nucleotides and then could contribute to the purinergic pathway activation. In PXE patients only the effects due to lack of PPi are observed (Uitto et al., 2011) as the amount of adenosine to activate purinergic receptors is low compared to that of pyrophosphate to prevent mineralization (Sachdeva and Gupta, 2013; Orris et al., 2016). However, we cannot exclude that the contribution of the ABCC6 in the regulation of the purinergic system can become more important in some liver diseases.

## Author contributions

FM carried out the experiments, FC prepared stably expressed HEK293 cells, MP performed western blot analysis, MFA and RM analyzed the data and performed statistical analysis, FB and AO designed experiments and wrote the paper. All authors read and approved the final manuscript.

### Conflict of interest statement

The authors declare that the research was conducted in the absence of any commercial or financial relationships that could be construed as a potential conflict of interest.

## Supplementary material

Supplemental Figure S1 Inhibition of efflux of doxorubicin by Probenecid. Doxorubicin efflux assay in HEK293 cells stably transfected with *ABCC6*-Flag-pcDNA in absence (•) or in presence (°) of 1 mM Probenecid and in HEK293 cells transfected with Flag-pcDNA in absence (▴) or in presence (Δ) of 1 mM Probenecid. Data are expressed as mean ± SD of three independent experiments. Significant differences at 30 min were determined by Student's *t*-test: ^*^*p*-value < 0.05, ^**^*p* < 0.01.

## Abbreviations

*ABCC6*: ATP-binding cassette, sub-family C, member 6
ACDC: arterial calcification due to deficiency of CD73
CD73: cluster of differentiation 73
ENPP1: ecto-nucleotide pyrophosphatase/phosphodiesterase type I
GACI: generalized arterial calcification of infancy
*NT5E*: ecto-5′-nucleotidase
PXE: pseudoxanthoma elasticum
TNAP: tissue non-specific alkaline phosphatase.
