# Supplementary figures and images for: Extracellular ATP Regulates CD73 and ABCC6 Expression in HepG2 Cells
Source: Front Mol Biosci. 2018 Aug 14;5:75. doi: 10.3389/fmolb.2018.00075 (PMC6102951; doi:10.3389/fmolb.2018.00075)

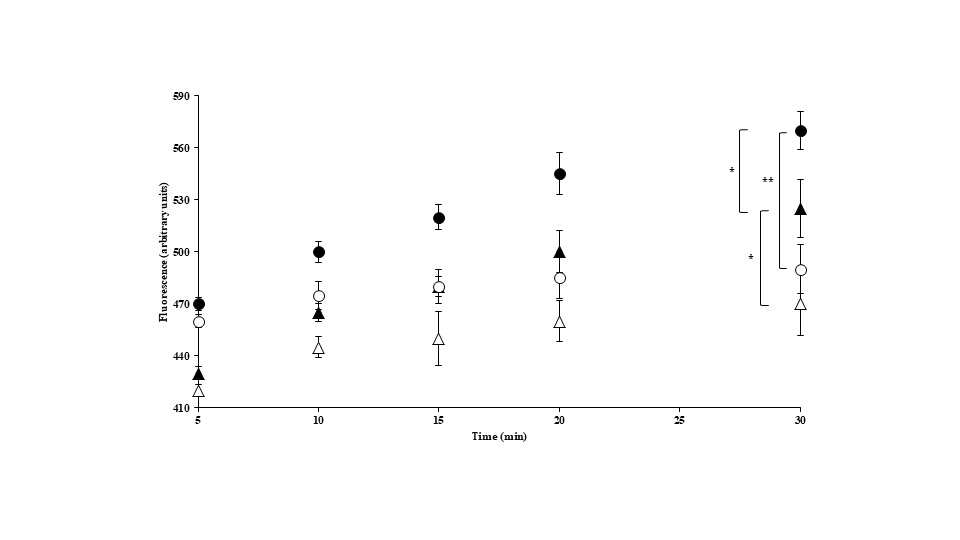

Supplement: Supplemental Figure S1 — Inhibition of efflux of doxorubicin by Probenecid. Doxorubicin efflux assay in HEK293 cells stably transfected with ABCC6-Flag-pcDNA in absence (•) or in presence (°) of 1 mM Probenecid and in HEK293 cells transfected with Flag-pcDNA in absence (▴) or in presence (Δ) of 1 mM Probenecid. Data are expressed as mean ± SD of three independent experiments. Significant differences at 30 min were determined by Student's t-test: *p-value < 0.05, **p < 0.01. [file Image_1.tif]
